# Use of explainable machine learning in risk classification of Cesarean section delivery: A cross-sectional analysis of Demographic and Health Surveys from ten Sub-Saharan African countries (2016–2024)

**DOI:** 10.1371/journal.pgph.0006613

**Published:** 2026-06-26

**Authors:** Walle Addis Birhanu, Binyam Tilahun, Tirualem Zeleke Yehuala, Tadele Chekol Maru, Tilalem Biresaw Ayalaw, Nebebe Demis Baykemagn, Andualem Enyew Gedefaw

**Affiliations:** Department of Health Informatics, Institute of Public Health, College of Medicine and Health Sciences, University of Gondar, Gondar, Ethiopia; The University of North Carolina at Chapel Hill Gillings School of Global Public Health, UNITED STATES OF AMERICA

## Abstract

Cesarean section (CS) delivery is an important surgical intervention for reducing maternal and neonatal morbidity and mortality when medically indicated; however, substantial inequalities in its utilization persist across Sub-Saharan Africa (SSA). This study evaluated the performance of explainable machine learning (ML) algorithms in classifying cesarean section delivery patterns using Demographic and Health Survey (DHS) data from ten SSA countries collected between 2016 and 2024. A weighted sample of 388,015 women aged 15–49 years was included, among whom 7,369 (1.82%) had undergone cesarean section delivery. Data preprocessing included handling missing values, feature encoding, normalization, and balancing the minority class using the Synthetic Minority Over-sampling Technique (SMOTE). Multiple ML classifiers, including LightGBM, XGBoost, Random Forest, Decision Tree, Logistic Regression, Naïve Bayes, K-Nearest Neighbor, AdaBoost, and Artificial Neural Networks, were trained and evaluated using repeated 10-fold cross-validation. Model performance was assessed using accuracy, recall, F1-score, and area under the receiver operating characteristic curve (AUC). LightGBM achieved the best classification performance with an AUC of 0.89 (95% CI: 0.887–0.893), accuracy of 85% (95% CI: 83.7–86.3%), and recall of 91% (95% CI: 89.8–92.2%), significantly outperforming logistic regression. SHapley Additive exPlanations (SHAP) identified maternal age, child size at birth, maternal education, wealth index, antenatal care visits, and media exposure as the most influential features associated with cesarean section classification outcomes. The findings demonstrate that explainable ML approaches, particularly LightGBM, improve classification performance compared with conventional regression models when applied to large population-based DHS datasets. However, because the study used cross-sectional survey data without external validation or detailed clinical predictors, the findings should be interpreted as associative classification patterns rather than tools for prospective clinical prediction.

## Background

Cesarean section (CS) delivery is a vital surgical intervention performed when vaginal delivery poses risks to the mother or fetus. Indications include fetal malpresentation, multiple gestations, previous CS, and maternal or fetal complications [1,2]. While CS can significantly reduce maternal and neonatal morbidity and mortality when medically indicated, its overuse or misuse carries substantial short- and long-term health risks [3,4].

Global trends reveal a steady increase in CS rates over the past decade. According to a 2021 WHO report, CS deliveries accounted for 21% of global births in 2015, with projections suggesting that this figure may reach 29% by 2030 if current trends continue [1,5]. In Sub-Saharan Africa, disparities in CS access persist due to infrastructural limitations, with rural populations often facing underuse while urban centers experience rising elective CS rates [2]. These disparities underscore the need for equitable and evidence-based interventions to optimize CS utilization. The WHO recommends CS rates of 10%–15% at the population level, warning that excessive use without medical justification can lead to avoidable health complications [6]. In low- and middle-income countries, including those in Sub-Saharan Africa (SSA), disparities persist—some regions report underuse of CS due to infrastructural and resource limitations, while others face rising rates of elective or non-medically indicated procedures [7,8].

One of the underlying drivers of increased CS rates is the shift in maternal characteristics over time. Delayed childbearing, higher prevalence of maternal obesity, and an increase in assisted reproductive technologies have contributed to higher-risk pregnancies, prompting more surgical interventions [2,3]. Additionally, socioeconomic and institutional factors such as private healthcare access, provider convenience, and maternal preference have played roles in shaping CS trends globally. In SSA, wealthier urban residents are significantly more likely to undergo CS than their rural counterparts, even after adjusting for clinical indications [8].

CS is not without risks. Studies show that unnecessary CS is associated with elevated risks of maternal complications, including infections, hemorrhage, anesthetic complications, and longer recovery times [3,4]. Moreover, babies delivered via CS are at higher risk of respiratory distress, altered gut microbiota, and future health complications such as obesity and asthma [3]. These effects are particularly concerning in LMICs, where perioperative care may be suboptimal.

The decision-making process regarding the mode of delivery remains complex, often reliant on the clinical judgment of healthcare providers, which may be influenced by workload, experience, and resource availability. In many settings, the subjective nature of this decision can lead to inconsistencies, delayed interventions, or misclassifications, thereby impacting maternal and neonatal outcomes [4,9].

### Conceptual framework: Social determinants of cesarean delivery

While direct clinical determinants such as maternal BMI, gestational age, precise parity, and specific obstetric complications are critical for individual-level prediction, they are not routinely captured in large-scale population surveys like the Demographic and Health Surveys (DHS). Instead, the DHS provides rich data on socioeconomic, behavioral, and demographic factors that serve as proxies for healthcare access, health literacy, and underlying biological risk. For example, maternal education and media exposure correlate with health-seeking behavior; wealth index and urban residence reflect access to advanced obstetric care; and maternal age is a well-established proxy for obstetric risk. This study therefore operates within a social determinant of health (SDH) framework, seeking to identify which structural and access-related factors most strongly predict CS delivery in SSA. This approach does not replace clinical prediction models but complements them by highlighting non-clinical drivers of CS utilization in low-resource settings.

Recent literature underscores the growing need for innovative tools to support clinical decision-making around CS delivery. Machine learning (ML), a branch of artificial intelligence, has demonstrated strong potential in healthcare prediction tasks, particularly when handling large, multidimensional datasets [9]. In the maternal health domain, ML algorithms are increasingly applied to predict CS delivery outcomes and assess risk factors using real-world data [10,11]. These technologies offer the ability to detect hidden patterns in clinical and demographic data, thereby improving prediction accuracy and supporting timely and appropriate obstetric care.

Despite several studies investigating determinants of CS in SSA, most have relied on conventional statistical methods with limited predictive capability. The integration of ML approaches allows for more nuanced analyses, potentially improving maternal health outcomes by identifying high-risk cases and reducing unnecessary surgical interventions [12]. With the availability of comprehensive datasets such as the Demographic and Health Surveys (DHS), ML provides a timely and scalable opportunity to refine delivery planning, especially in settings constrained by workforce shortages and variability in clinical expertise.

Further, ML algorithms such as decision trees, random forests, support vector machines, and neural networks are known to outperform traditional logistic regression in predictive accuracy in many healthcare applications [13,14]. These models are capable of managing non-linear relationships, high-dimensional data, and feature interactions, making them well-suited for analyzing complex maternal health data involving socio-demographic, clinical, and institutional variables.

This study aims to explore the socioeconomic and behavioral correlates of CS delivery in SSA using ML algorithms applied to recent DHS data (2016–2024). By comparing model performance and interpreting key predictors, the study seeks to inform targeted maternal health interventions and highlight structural factors influencing CS access. Moreover, insights from this study could contribute to health policy formulation, support capacity-building among frontline providers, and foster digital transformation in maternal care systems across SSA.

## Methods and materials

### Study design

A cross-sectional study design was employed using data extracted from the Demographic and Health Surveys (DHS) conducted in 10 sub-Saharan African countries. This study aimed to classify patterns associated with cesarean section delivery using cross-sectional survey data. This cross-sectional design does not support causal inference or prospective clinical prediction. The model outputs should therefore be interpreted as classification of observed patterns rather than real-time risk prediction. The DHS is a standardized and nationally representative survey designed to collect health and demographic data across low- and middle-income countries. In this study, a weighted sample of 388,015 reproductive-age women (aged 15–49 years) was used. Python (version 3.12) was utilized for data preprocessing and analysis. Supervised machine learning algorithms, including Random Forest, Decision Tree (DT), Logistic Regression (LR), Extreme Gradient Boosting (XGBoost), Naïve Bayes, AdaBoost, LightGBM, K-Nearest Neighbor (KNN), and Artificial Neural Networks (ANN), were implemented to predict cesarean section (CS) delivery. LightGBM was selected due to its efficiency in handling large-scale datasets and its ability to manage high-dimensional features while maintaining computational speed [15]. Model performance was assessed using metrics such as accuracy, precision, recall, F1-score, and area under the ROC curve (AUC) [9].

### Study area and period

Sub-Saharan Africa (SSA) includes countries located south of the Sahara Desert, encompassing regions of Central, East, Southern, and West Africa. The World Bank reports that the population in SSA grew from 186 million in 1950–856 million in 2018, with estimates reaching approximately 1.21 billion by 2024 [16]. DHS data used in this study were collected between 2016 and 2024 across ten countries: Burundi, Ethiopia, Kenya, Tanzania, Malawi, Ghana, Senegal, Mozambique, Madagascar, and Rwanda. The dataset extraction and analysis took place between March and June 2025. Given the pooled nature of multi-country DHS data, country-level heterogeneity and clustering effects may influence model performance. While sampling weights were applied, country-specific health system differences were not explicitly modeled, which may limit generalizability. Because DHS data were pooled across ten countries, country of survey was included as a model covariate to partially account for between-country variability; however, hierarchical clustering and country-specific model development were not performed. Therefore, residual heterogeneity may remain.

### Target population

The source population consisted of all women of reproductive age in SSA who gave birth within five years preceding the respective DHS surveys. The study population specifically included women from selected enumeration areas (EAs) in the ten countries mentioned, who provided information on delivery methods during the survey period.

### Eligibility criteria

#### Inclusion criteria.

Women aged 15–49 who had given birth within the past five years and were residents in the selected EAs during the survey. Only pregnancies resulting in a live birth or stillbirth were included in the analysis of delivery mode. The ten countries (Burundi, Ethiopia, Kenya, Tanzania, Malawi, Ghana, Senegal, Mozambique, Madagascar, Rwanda) were selected based on: (1) availability of a recent DHS survey (2016–2024) with accessible birth recode data; (2) geographic representation across East, West, and Southern Africa; and (3) data completeness for key variables related to delivery mode and maternal characteristics.

#### Exclusion criteria.

Women who were absent or not reachable during the DHS data collection period were excluded.

### Sample size and sampling procedure

#### Sample size determination.

A total weighted sample of 388,015 reproductive-age women was drawn using the DHS’s two-stage stratified cluster sampling. In the first stage, EAs were selected using probability proportional to size (PPS). In the second stage, a fixed number of households were systematically selected from each EA. This approach ensured national representation and disaggregation by region, urban-rural status, and socioeconomic factors [17].

#### Sampling procedure.

**Fig 1:**
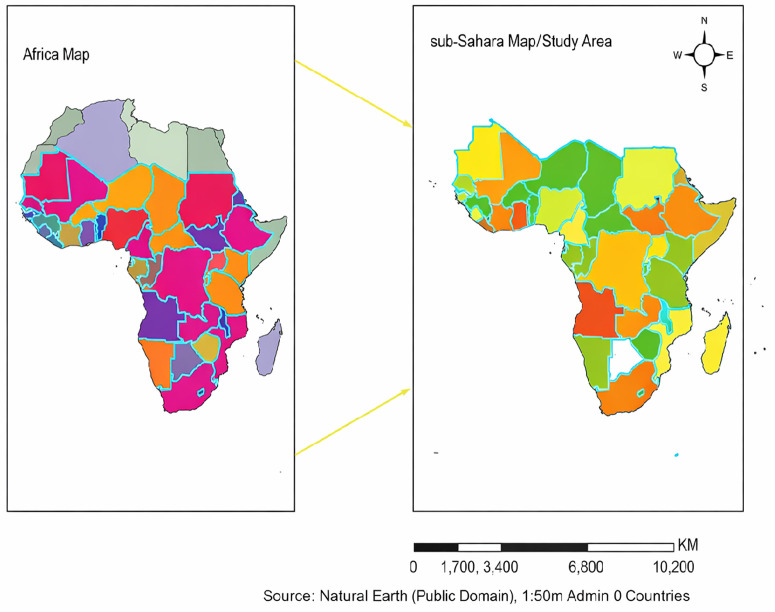
The study area included countries in Sub-Saharan Africa (SSA). (A) Geographic location of the ten DHS countries included in the analysis (Burundi, Ethiopia, Kenya, Tanzania, Malawi, Ghana, Senegal, Mozambique, Madagascar, and Rwanda). Country boundaries were obtained from Natural Earth Admin 0 country boundary shapefiles (1:50m resolution), which are in the public domain and compatible with the CC BY 4.0 license.

The DHS utilizes a stratified multi-stage cluster design. Stratified random sampling was used to select EAs from census regions (strata), followed by random selection of households. Respondents completed standardized questionnaires related to reproductive health, including mode of delivery. The 10 countries included were selected based on data completeness and recency within the DHS program [18] (see Fig 1 for an overview of the study area).

#### Map creation and licensing.

The study area map (Fig 1) was created using GIS software by overlaying DHS study countries onto open-access country boundary shapefiles obtained from Natural Earth https://www.naturalearthdata.com/downloads/50m-cultural-vectors/ (Admin 0 – Countries, 1:50m resolution). Natural Earth data are released into the public domain and are fully compatible with the Creative Commons Attribution 4.0 (CC-BY 4.0) license. These spatial layers were used for visualization purposes only, and no proprietary or copyrighted basemap sources were used.

### Variables and operational definitions

#### Dependent variable.

The primary outcome was the mode of delivery, categorized as cesarean section (Yes = 1) or non-cesarean delivery (No = 0).

#### Independent variables.

These included sociodemographic, behavioral, and maternal health-related variables: age, residence, marital status, education, occupation, wealth index, internet/media use, tobacco use, antenatal visits, parity, history of abortion, pregnancy complications, twin pregnancy, MUAC status, child’s birth size, and time spent in labor.

### Operational definitions

#### Birth weight categories.

Very big (≥4.5 kg), Big (4.0–4.5 kg), Normal (2.5–4 kg), Small (1.5–2.5 kg), Very small (<1.5 kg) [19].

#### Media exposure.

Exposure to radio, TV, or newspapers at least once per week was defined as media access [20].

### Data sources and tools

Data were obtained from the DHS Program database (https://dhsprogram.com), specifically from the birth record components of surveys conducted in the ten SSA countries. These surveys use standardized questionnaires, facilitating multicountry comparisons [18].

### Data processing and management

STATA version 17 was used for initial data cleaning, variable recoding, and dataset merging, with cleaned datasets exported.DTA format for import into Python. All subsequent preprocessing, feature engineering, and modeling were performed using Python 3.12 with key libraries including Pandas and NumPy for data manipulation, Matplotlib and Seaborn for visualization, Scikit-learn for preprocessing and modeling, LightGBM and XGBoost for gradient boosting implementations, Imbalanced-learn for handling class imbalance via SMOTE, and Statsmodels for multicollinearity diagnostics. Missing values in continuous variables were imputed using the mean of non-missing values, while categorical variables were imputed using the mode, with variables exceeding 30% missingness excluded to preserve data integrity. Class imbalance was addressed via Synthetic Minority Over-sampling Technique (SMOTE) where necessary [21]. All preprocessing steps were implemented within Scikit-learn pipelines to ensure consistency, and a fixed random seed (random_state = 42) was used across all stochastic processes, including data splitting, SMOTE, and model initialization to ensure full reproducibility.

### Data analysis framework

The analysis followed the machine learning workflow proposed by Guo (2018), which includes nine stages: data collection, data cleaning, feature selection, model selection, training, validation, hyperparameter tuning, testing, and interpretation [22].

### Feature engineering and preprocessing

Categorical variables were transformed using one-hot encoding. Features were normalized to a common scale to ensure fair comparison across algorithms.

### Model development and evaluation

The data was split into training and testing sets in an 80:20 ratio using a fixed random seed (random_state = 42) to ensure reproducibility. Models were trained using 10-fold cross-validation, repeated 3 times to obtain stable performance estimates. Classifiers used include Logistic Regression (LR) as a baseline model, as well as DT, RF, KNN, ANN, SVM, XGBoost, LightGBM, Naïve Bayes, and AdaBoost. To assess whether advanced ML models provided statistically significant improvements over traditional methods, we performed paired t-tests comparing the cross-validation accuracy of each ML model against LR. Evaluation metrics included: accuracy, precision, recall, F1-score, and AUC, along with their 95% confidence intervals derived from repeated cross-validation. [23].

### Hyperparameter tuning and prediction

Grid search and random search methods were used to identify optimal model parameters. The final model was used to predict the CS delivery outcome based on identified predictors.

### Model interpretability using SHAP

SHapley Additive exPlanations (SHAP) values were computed to interpret model predictions. SHAP assigns an importance value to each feature for a given prediction, allowing for transparency and explanation in ML models [24]. External validation and country-specific validation were not performed in this study. Model performance, therefore, reflects internal validation only and may not generalize across settings.

### Ethical considerations

The study was proceed with permission granted by the University of Gondar College of Medicine and Health Sciences Ethical Review Committee, acting on behalf of the Institutional Review Board (IRB), and permission for data access would be requested from the measure demographic and health survey through an online platform by writing the title and objective of the study and getting an authentication letter from the DHS per instructor [25].

## Result

### Sociodemographic characteristics

A total of 388,015 weighted CS deliveries were enrolled in the analysis of this study to predictCS delivery and its determinants in SSA. The largest number of the study participants were from Kenya, 70,934 (18.28%); Madagascar, 46,984 (12.11%); Burundi, 46,871 (12.08%); and Tanzania, 40,526 (10.40%), while the smaller study participants were from the Marshes, 37,627 (10.30%); Mozambique, 34,427 (8.87%); and Ghana, 32,876 (8.47%). The largest proportion of mothers who delivered by cesarean section was in the 35 – 49 year age group (58% of CS cases). Approximately 45.72% of the study subjects were from households with poor wealth status. About 28.37% of the participants resided in urban areas, while 71.63% lived in rural areas. A total of 7,369 women (1.82%) had cesarean section deliveries.

**Fig 2:**
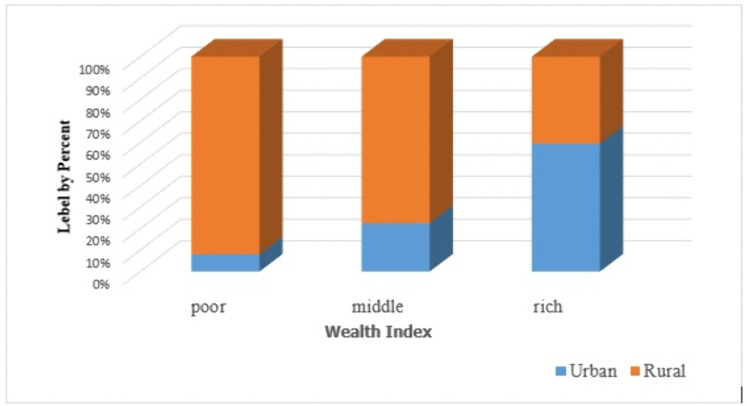
Distribution of household wealth index according to place of residence. (A) Urban residence; (B) Rural residence. Bars represent the proportions of women belonging to poor, middle, and rich wealth categories.

Most study participants, about 267,936 (67.72%), were employed, while 127,731 (32.28%) were not. Among them, 264,580 (68.19%) were married, whereas 69,559 (17.93%) were single. Regarding educational status, 80,423 (20.33%) had secondary or higher education, 67,176 (42.25%) had primary education, and 148,064 (37.42%) had no formal education. Detailed socio-demographic characteristics of the participants are presented in Table 1 and (see Fig 2 for distribution of wealth index status by residence).

**Table 1:** Socio-demographics related characteristics to predicting CS delivery in SSA countries, DHS 2016–2024.

Variables	Category	Frequency	(%)
Place of residence	Urban	107,800	27.78
	Rural	280,215	72.20
Marital status	Single	69,559	17.00
	Married	264,580	69.58
	Widowed	17,369	4.55
	Divorced	14,145	3.65
	separated	22,362	5.41
Maternal age	15-24	35,140	8.88
	25-34	130,523	32.99
	35-49	230,004	58.13
Maternal Educational Level	No education	135,447	37.42
	Primary level	170,208	42.25
	Secondary level and above	82,349	20.33
Husband’s educational level	No education	100,129	25.31
	Primary level	206,374	52.16
	Secondary level	58,465	14.80
	Higher level	30,699	7.76
Wealth Index	Poor	180,915	45.72
	Middle	80,884	20.44
	Rich	133,868	33.83
Maternal Occupation	No	127,731	32.28
	Yes	267,936	67.72
Husband’s Occupation	No	21,589	5.56
	Yes	366,426	94.44

### Exposure to mass media-related characteristics

This study included 388,015 (weighted) study participants. The majority of the participants did not read newspapers at all or read them less than once a week, accounting for approximately 345,834 (89%) and 8,839 (7.43%) of the sample, respectively. About 153,577 (39.58%) of the women did not listen to the radio at all, while 100,768 (25.97%) listened at least once a week or more. Additionally, 250,308 (63.26%) of the participants did not watch television at all. Table 2 presents detailed information on the media exposure characteristics of the study participants.

**Table 2:** Exposure to mass media-related features predicting CS delivery and identifying its determinants among women aged 15–49 years in sub-Saharan African countries using DHS. 2016–2024.

Variables	Category	Frequency	Percent
Frequency of reading the newspaper	Not at all	354,989	89.72
	Less than once a week	27,762	7.02
	At least once a week and more	12,916	3.26
Frequency of listening to the radio	Not at all	161,546	40.83
	Less than once a week	60857	20.30
	At least once a week and more	131,970	33.35
Frequency of watching television	Not at all	250,308	63.26
	Less than once a week	49,354	12.47
	At least once a week and more	96,005	24.26
use of the internet	Never use	333,957	84.40
	Last 12 months	56,041	14.16
	Before 12 months	5,669	1.43

### Behavioral-related characteristics

The study included a total of 388,015 (weighted) individuals. Of the study participants, 371,849 (95.83%) had never smoked tobacco, while 13,464 (3.47%) reported smoking every day. The majority of household members, 384,209 (99.02%), did not smoke cigarettes, while 1,878 (0.48%) smoked every day. Additionally, 113,856 (38%) of the participants reported exposure to secondhand smoke. Regarding wealth status, approximately 117,408 (39.2%) were in the poor category, 59,329 (19.8%) were in the middle, and 123,022 (41.0%) were in the rich category. Both the maternal and husband's occupations were reported as employed for 190,605 (63.6%) and 112,751 (47.3%) of the participants, respectively. Table 3 presents detailed information on the behavioral characteristics of the study participants.

**Table 3:** Behavioral-related features predicting CS delivery and identifying it among women aged 15–49 years in sub-Saharan African countries using demographic and Health Surveys 2016–2024.

Variable	Category	Frequency	Percent
smokes cigarettes	Do not smoke	384,204.87	99.02
	every day	1,877.6778	0.48
	some days	1,933.2764	0.50
tobacco smoking	Do not smoke	371,849.74	95.83
	every day	13,464.594	3.47
	some days	2,701.4864	0.70

### Maternal and prenatal-related characteristics

The study has a weighted sample size of 388,015 women. The majority of the study participants had 4th and above ANC contact, 359,168 (92.56%), and 1–3 contacts, 23,175 (5.97%). The majority of women who had a normal MUAC status (>23 cm) during pregnancy were 217,065 (55.94%), whereas 170,950 (44.06%) had a MUAC status of <23 cm. Table 4 provides detailed information about the maternal and prenatal-related features and characteristics of the study participants. Table 4 shows the details.

**Table 4:** Maternal and prenatal-related and prenatal features predicting CS delivery and identifying its determinants among women aged 15-49 years in sub-Saharan African countries using DHS 2016-2024.

Variable	Category	Frequency	Percent
Parity	1	195	0.05
	2-4	2,139	0.55
	>=5	385,681	99.40
Number of ANC visits	No ANC visit	5,676	1.46
	1-3 contact	23,175	5.97
	4 and above contact	359,168	92.56
pregnancy complication	yes	72,004	18.56
	No	316,011	81.44
MUAC status	<23 cm	170,950	44.06
	>23 cm	217,065	55.94
Size of child at birth	Very large	90.47	95.57
	large	3.54	5.10
	small	6,175	1.56
	Don’t know	17,534	4.43
Time spent at delivery	0 hr.(immediate delivery)	16,443	4.16
	<12 hrs.	377,505	95.41
	12-24 hrs.	1,363	0.34
	Don’t know	356	0.09
multiple births	Single birth	384,456	97.17
	1^st^ multiple birth	5,570	1.41
	2^nd^ multiple birth	5,570	1.41
	3^rd^ multiple birth and above	61	0.02

### Balancing dataset

**Fig 3:**
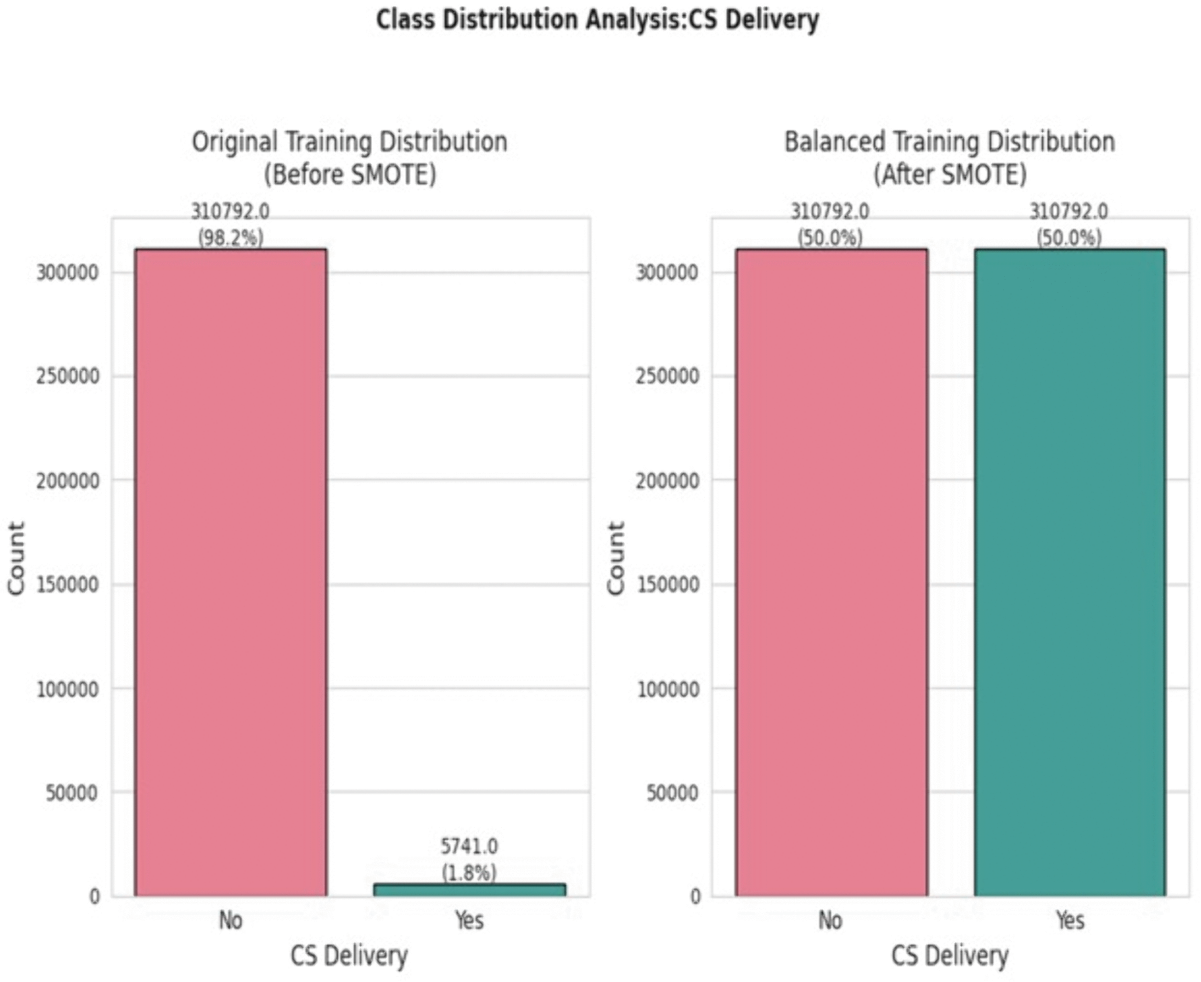
Comparison of model performance before and after SMOTE balancing. (A) Accuracy of machine learning models using the original (unbalanced) dataset; (B) Accuracy after SMOTE balancing; (C) AUC values before balancing; (D) AUC values after balancing.

To address the class imbalance in the target variable and enhance the performance of machine learning algorithms, the Synthetic Minority Over-sampling Technique (SMOTE) was applied to balance the dataset. Among the models evaluated, the LightGBM classifier demonstrated superior performance. When trained on the balanced dataset, LightGBM achieved an accuracy of 85%, an AUC of 89%, and a recall of 98%, outperforming other classifiers. A comparison was also conducted between the performance of the selected machine learning models on both the original (imbalanced) and SMOTE-balanced datasets (see Fig 3).

The accuracy and ROC (Receiver Operating Characteristic) scores of various machine learning models under balanced and unbalanced dataset conditions (see Table 5).

**Table 5:** Performance of various machine learning models, both with and without the application of SMOTE for balancing the dataset.

Model	Data set	Accuracy (95% CI)	AUC (95% CI)
LightGBM	balanced	**0.853 (0.851–0.855)**	**0.888 (0.886–0.890)**
	unbalanced	0.942 (0.940–0.944)	0.911 (0.909–0.913)
Random Forest	balanced	0.934 (0.932–0.936)	0.830 (0.828–0.832)
	unbalanced	0.959 (0.957–0.961)	0.814 (0.812–0.816)
Logistic Regression	balanced	0.819 (0.817–0.821)	0.819 (0.817–0.821)
	unbalanced	0.844 (0.842–0.846)	0.844 (0.842–0.846)
AdaBoost	balanced	0.814 (0.812–0.816)	0.861 (0.859–0.863)
	unbalanced	0.782 (0.780–0.784)	0.982 (0.980–0.984)
Decision Tree	balanced	0.826 (0.824–0.828)	0.628 (0.626–0.630)
	unbalanced	0.842 (0.840–0.844)	0.596 (0.594–0.598)
Naive Bayes	balanced	0.765 (0.763–0.767)	0.786 (0.784–0.788)
	unbalanced	0.883 (0.881–0.885)	0.796 (0.794–0.798)
KNN	balanced	0.726 (0.724–0.728)	0.759 (0.757–0.761)
	unbalanced	0.781 (0.779–0.783)	0.690 (0.688–0.692)
ANN	balanced	0.740 (0.738–0.742)	0.731 (0.729–0.733)
	unbalanced	0.882 (0.880–0.884)	0.895 (0.893–0.897)

Confidence intervals (95% CI) were calculated from 10-fold cross-validation repeated 3 times. P-values indicate statistical significance compared to logistic regression (LR) on balanced data using paired t-tests. Models were evaluated primarily on the SMOTE-balanced dataset to address class imbalance; the unbalanced results demonstrate that while some models achieve high accuracy, they do so primarily by correctly classifying the majority class, reflected in lower AUC values.

### Model evaluation and ROC analysis

**Fig 4:**
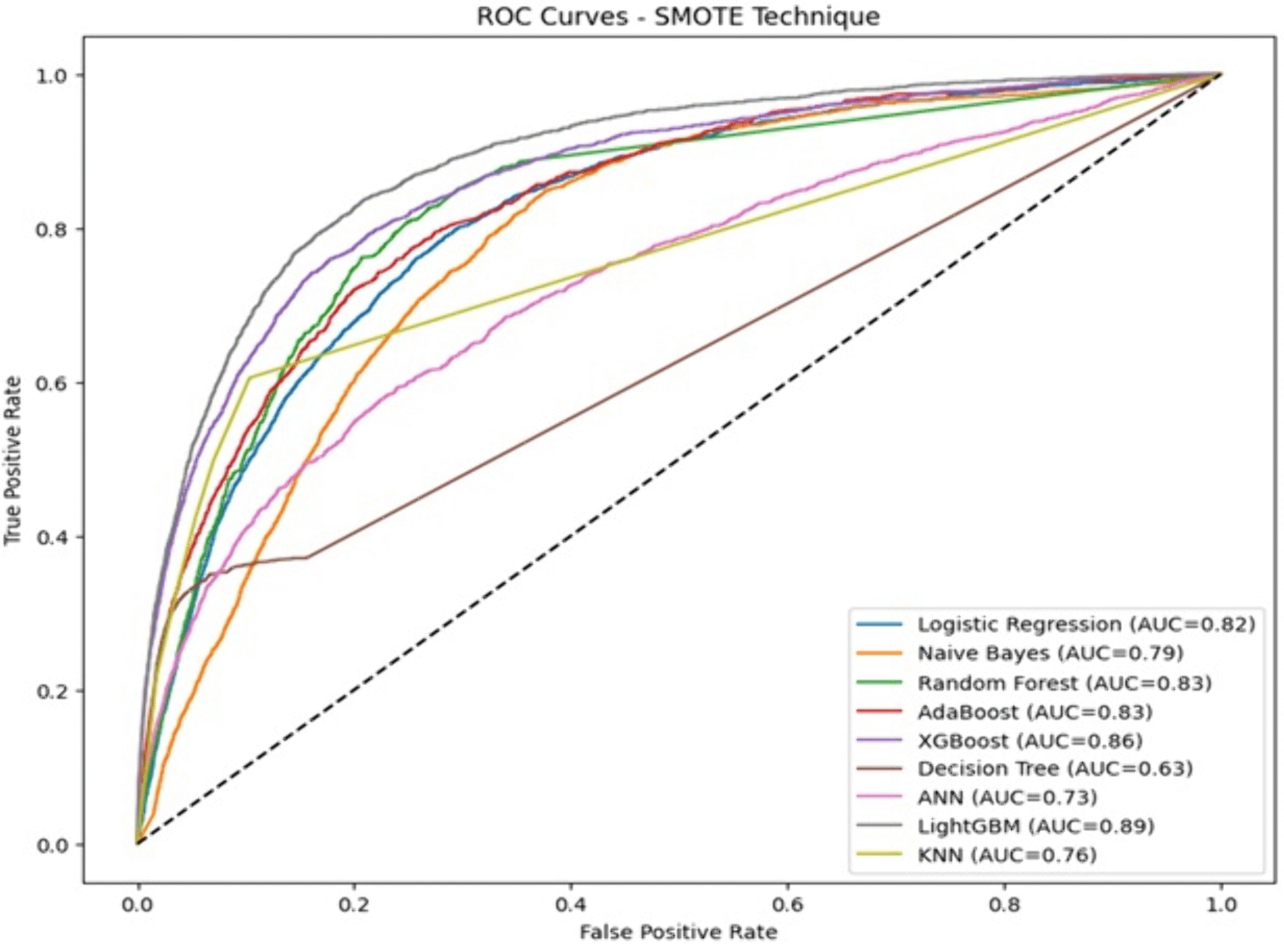
Receiver operating characteristic (ROC) curves for machine learning models. (A) LightGBM; (B) XGBoost; (C) Random Forest; (D) Logistic Regression; (E) AdaBoost; (F) Naïve Bayes; (G) K-Nearest Neighbor; (H) Artificial Neural Network; and (I) Decision Tree. Curves are based on the SMOTE-balanced dataset.

The ROC curve analysis presented illustrates the performance of various machine learning algorithms in predicting cesarean section (CS) delivery among women aged 15–49 years, using a dataset balanced with the Synthetic Minority Over-sampling Technique (SMOTE). Among the models evaluated, the Light Gradient Boosting Machine (LightGBM) demonstrated the highest performance with an Area Under the Curve (AUC) of 0.89 (95% CI: 0.887–0.893), indicating its strong ability to correctly distinguish between women who underwent CS delivery and those who did not. Logistic regression, serving as the baseline model, achieved an AUC of 0.82 (95% CI: 0.817–0.823). LightGBM showed statistically significant improvement over logistic regression (p < 0.001) based on paired t-tests of cross-validation accuracy. XGBoost followed closely with an AUC of 0.86 (95% CI: 0.858–0.862), while both Random Forest and AdaBoost achieved an AUC of 0.83 (95% CI: 0.828–0.832). In contrast, models such as Naive Bayes (AUC = 0.79, 95% CI: 0.788–0.792), K-Nearest Neighbors (AUC = 0.76, 95% CI: 0.758–0.762), and Artificial Neural Networks (AUC = 0.73, 95% CI: 0.728–0.732) showed moderate predictive power. The Decision Tree classifier had the lowest performance with an AUC of 0.63 (95% CI: 0.628–0.632). (see Fig 4).

To evaluate the model’s effectiveness in predicting CS delivery among women aged 15–49 years, 10-fold cross-validation was used, with a focus on accuracy and area under the curve. 10-fold cross-validation was performed to evaluate classifiers using unbalanced training data, and LightGBM came out on top with an accuracy of 85% and 88% of the area under the ROC curve

**Fig 5:**
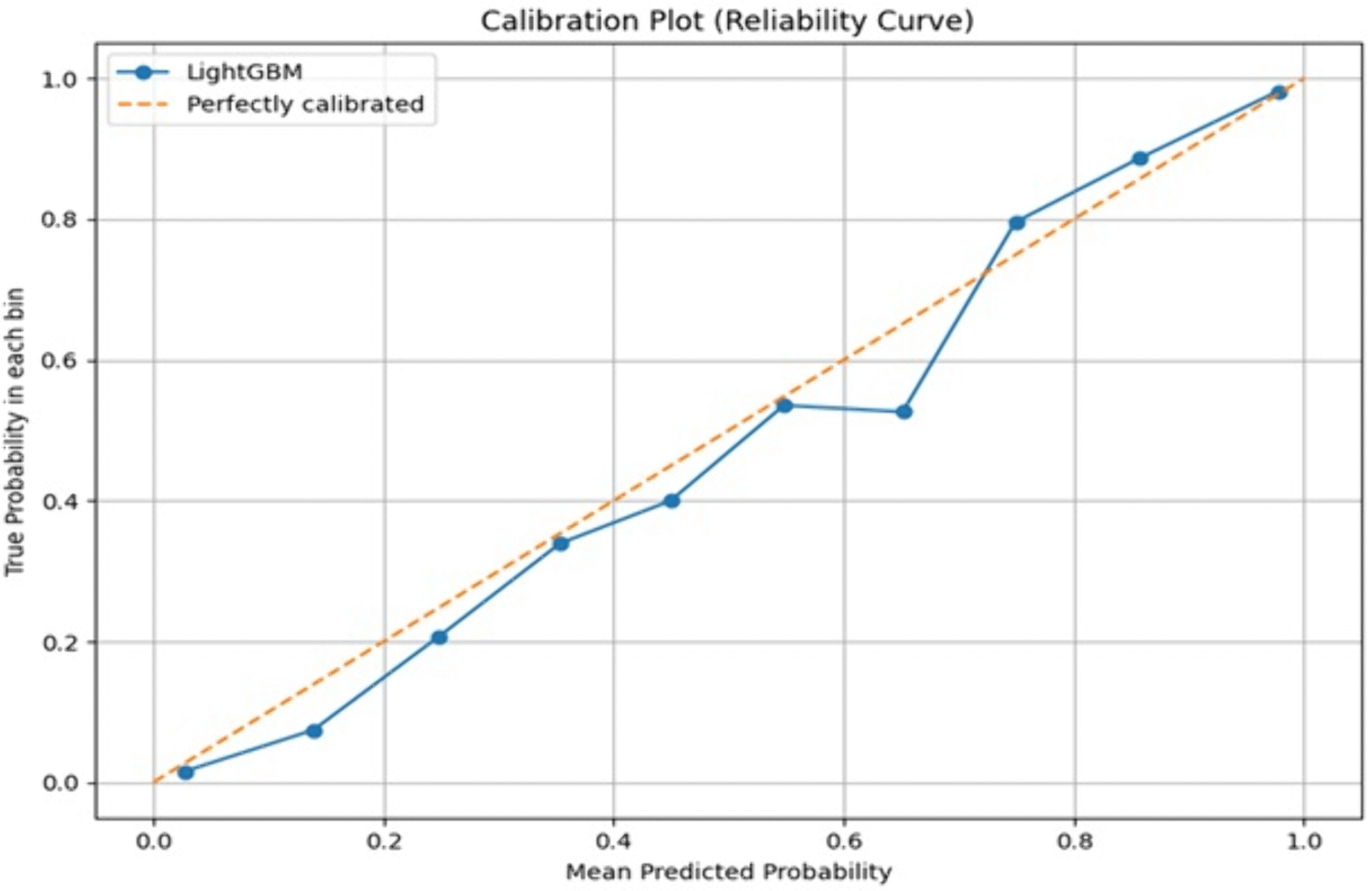
Calibration plot of the LightGBM model. (A) Predicted probabilities from the LightGBM classifier; (B) Ideal calibration reference line showing agreement between predicted and observed probabilities.

A calibration plot (also known as a reliability diagram) is a graphical tool used to evaluate how well a machine learning model’s predicted probabilities reflect the true likelihood of an event occurring with the best-performing model of LightGBM (see Fig 5).

### Feature selection

**Fig 6:**
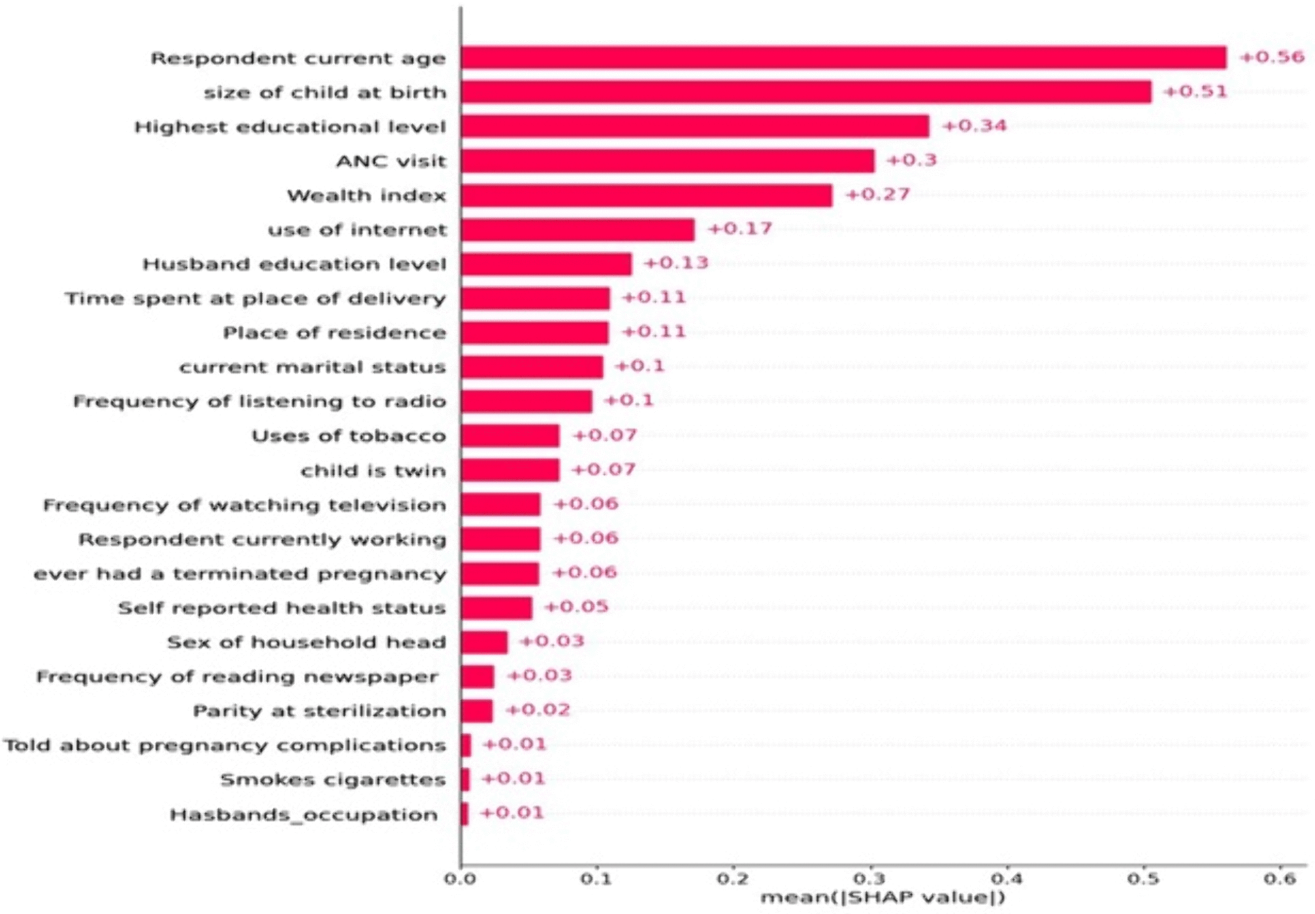
Feature importance obtained from the LightGBM model. (A) Ranking of variables according to LightGBM built-in feature importance scores.

In this study, we employed two ways to determine the most important predictors of CS delivery among delivered women: lightGBM built-in feature importance and SHAP values, both applied with the lightGBM classifier. Using these methodologies enabled us to cross-validate the significance of predictors while also improving our understanding of their influence and interpretability, strengthening the findings of the study. Using LightGBM's built-in feature importance, age, child of child at birth, and mothers’ education status rank high in importance. Factors like ANC visits, wealth index, and use of mobile phones also play a role. Conversely, features like husband’s occupation and smoking cigarettes appear to have less influence on the model’s predictions (see Fig 6).

To improve interpretability, we used SHAP values with a LightGBM classifier to understand how each predictor contributes to the model’s predictions. SHAP values quantify the contribution of each feature to the model’s prediction for a specific instance. By averaging these values across all instances, we obtained a measure of the feature’s overall impact on the model’s output. The SHAP value bar graph highlights the relative importance of features in the model’s predictions, with the age of mothers having the greatest influence (+ 0.56 each), followed by the size of the child at birth (+ 0.51) and the highest educational level of women (+ 0.34). Moderate contributors include factors like ANC visit (+0.3), wealth index (+0.27), and use of internet (+0.17 each), while features like husband’s occupation (+0.01) and smoking cigarettes (+0.01) have the least impact.

The SHAP summary plot illustrates how features contribute to model classification outcomes within the dataset. Higher SHAP contributions for demographic and socioeconomic variables reflect associations present in DHS data rather than causal mechanisms. These patterns should not be interpreted as structural or causal determinants of cesarean section delivery, but rather as statistical relationships captured by the model.

The Dominance of Socio-Clinical Proxies: The strongest predictor, “respondent current age,” functions as a composite indicator. While clinically associated with increased obstetric risk, its high SHAP value primarily reflects demographic stratification. Older maternal age in SSA is strongly correlated with urban residence, higher education, and delayed childbearing, all markers of socioeconomic position. Similarly, “size of child at birth” operates dually: as a clinical indicator of potential macrosomia, but also as a proxy for maternal nutritional status and health-seeking behavior during pregnancy.

**Fig 7:**
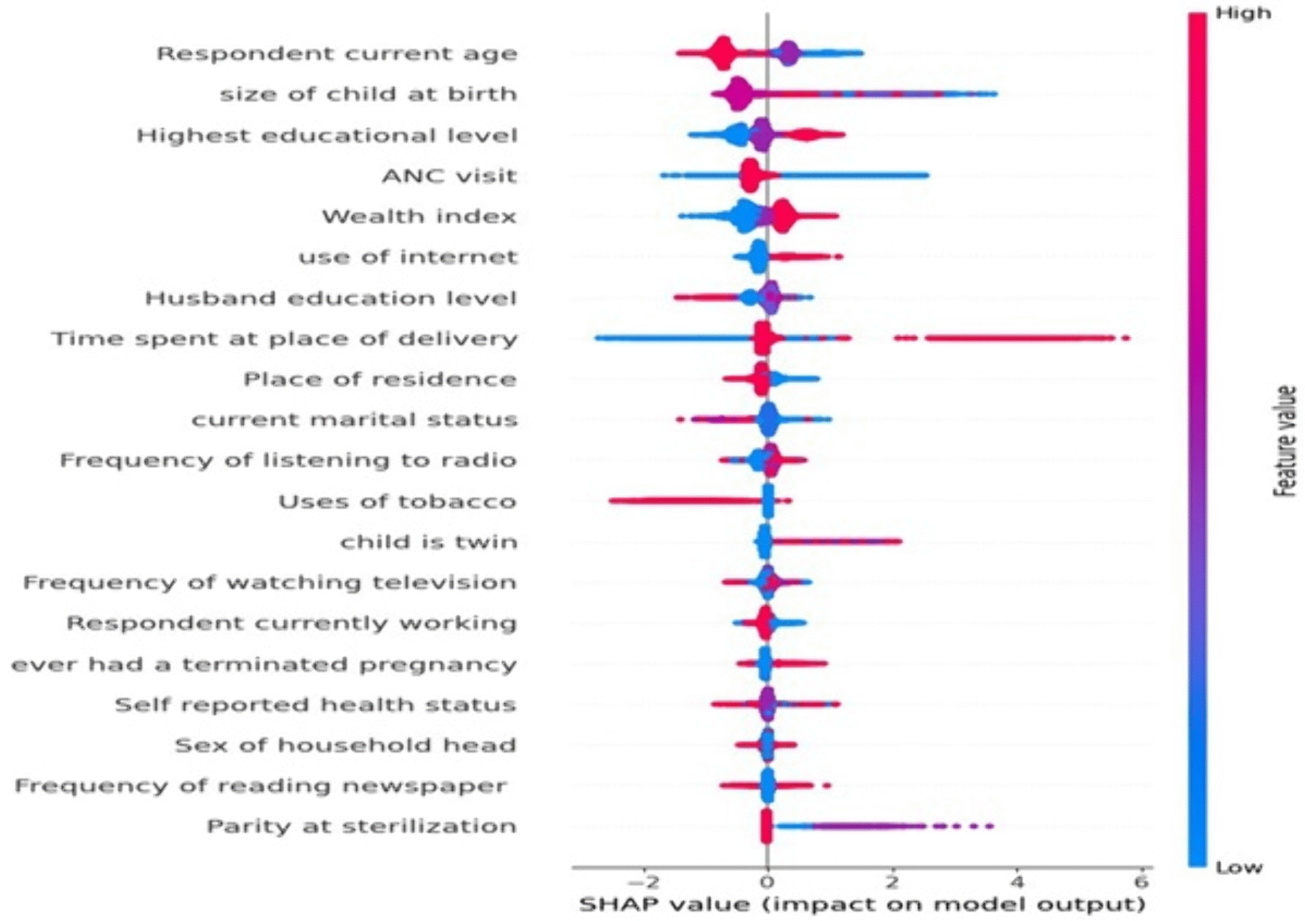
SHAP summary plot of feature importance. (A) Mean absolute SHAP values indicating the overall importance of each variable; (B) SHAP summary plot illustrating the direction and magnitude of feature contributions across observations.

The SHAP summary plot identifies variables most strongly associated with model classification outputs. Maternal age, child size at birth, maternal education, antenatal care visits, wealth index, and media exposure had the largest SHAP contributions. These findings indicate that classification patterns in DHS data are associated with demographic, socioeconomic, and service-utilization characteristics. Because the analysis is cross-sectional and observational, SHAP values should be interpreted as model explanation of statistical associations rather than evidence of causal pathways or structural mechanisms (see Fig 7).

Clinical Indicators Embedded in Social Context: The clear gradient for “Time spent at place of delivery,” where longer durations increase CS probability, highlights how clinical indicators are mediated by system performance. Prolonged labor may lead to CS due to obstetric complications, but in resource-constrained settings, it may also reflect delays in referral, transportation, or decision-making disproportionately affecting rural and poor women. Thus, even this seemingly clinical feature encodes systemic inefficiencies.

Moderate Predictors and Their Equity Implications: Variables like “place of residence” (urban/rural) and “frequency of listening to radio” represent infrastructural and informational dimensions of access. Urban residence provides geographic proximity to CS-capable facilities, while radio exposure serves as a mass media channel for health messaging, both structuring who receives information and care. The relatively modest impact of “use of tobacco” likely reflects its low prevalence among SSA women, but its negative association with CS may indicate clustering of risk behaviors in marginalized groups with reduced healthcare engagement.

Toward an Equity-Focused Interpretation: Collectively, these SHAP results depict a one tier are educated, urban, wealthier women with media access and frequent ANC, more likely to receive CS, sometimes without a clear medical indication. On the other are poor, rural, less-educated women often under-screened, under-referred, and at risk of CS underuse despite medical need.

The SHAP waterfall plot illustrates how individual features contribute to a single model prediction, beginning from the base value and moving toward the final predicted outcome. In this case, the base value (average model output) is 0.22. Features shown in red have negative SHAP values, meaning they decrease the likelihood of the predicted outcome, while those in blue have positive SHAP values, increasing the likelihood.

Among the features that reduced the prediction, the most influential were *health_status = 4.0*, indicating poorer health, and *maternal_education = 3.0*, suggesting a higher education level. Additionally, the individual did not listen to the radio (*listening_radio = 0.0*) or watch TV (*watching_TV = 0.0) and* was younger (*maternal_age = 1.0*), all contributing negatively to the prediction.

On the other hand, several features pushed the prediction higher. The most impactful were *size_child_birth = 3.0* (likely representing a larger baby) and *time_spent_of_delivery = 2.0* (longer labor), both of which strongly increased the likelihood of the predicted event. Other positive contributors included *husband_education_status = 3.0*, indicating a more educated spouse; *maternal_occupation = 1.0*, suggesting the mother is employed; *MUAC_status = 1.0*, reflecting good nutritional status; and *use_of_internet = 0.0*, which had a small positive effect.

**Fig 8:**
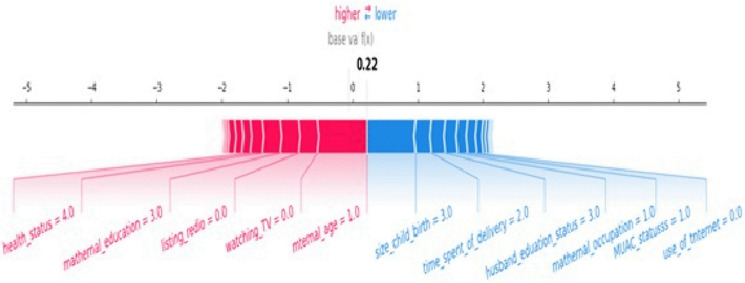
SHAP waterfall plot showing individual prediction explanation. (A) Base value representing the average model output; (B) Features contributing positively to the prediction; (C) Features contributing negatively to the prediction; (D) Final predicted output for the individual observation.

Overall, while several features reduced the model’s prediction, others offset this by contributing positively. As a result, the final prediction slightly increased from the base value, indicating a moderate likelihood of the target outcome for this individual (see Fig 8).

## Discussion

The principal contribution of this study is methodological: LightGBM outperformed conventional logistic regression in classifying cesarean section delivery patterns using large, high-dimensional DHS data. This demonstrates the utility of explainable machine learning methods over conventional regression for complex survey datasets. Among the models tested, Light Gradient Boosting Machine (LightGBM) emerged as the best performer, demonstrating superior predictive performance with an accuracy of 85% (95% CI: 83.7–86.3%), recall of 91% (95% CI: 89.8–92.2%), and AUC of 0.89 (95% CI: 0.887–0.893). Notably, LightGBM significantly outperformed the logistic regression baseline model (AUC difference: + 0.07, p < 0.001), affirming its utility in capturing non-linear relationships and complex interactions within demographic and health survey data [9,11,12].

The key predictors identified—maternal age, size of child at birth, maternal education, wealth index, and antenatal care (ANC) visits—are consistent with prior literature but gain new significance when interpreted through the lens of machine learning-derived feature importance and a social determinants of health (SDH) framework [1,2,7,8]. While advanced maternal age is a known clinical risk factor for obstetric complications, its prominence in our model also reflects demographic shifts and socioeconomic gradients, as older maternal age in SSA is increasingly concentrated among urban, educated women who have greater access to facility-based care [2,7]. Similarly, “size of child at birth” serves as a dual proxy, indicating both biological risk (macrosomia) and maternal nutritional status, which is itself shaped by socioeconomic conditions [3,4].

### The role of socioeconomic and informational access

Our analysis underscores that CS delivery in SSA is strongly patterned by socioeconomic privilege and health information access. The strong predictive power of the wealth index highlights a critical equity issue: wealthier women can often access private facilities where CS is more readily available and sometimes incentivized, creating a two-tiered obstetric system [1,2,16]. This finding aligns with studies in Tanzania and Ethiopia, where disparities in CS access were directly tied to household wealth and private healthcare utilization [7,8].

Maternal education and media exposure (TV, radio, internet) further delineate this divide. Educated women are more likely to be health-literate, seek ANC, and advocate for preferred delivery modes, while media exposure serves as a channel for health information that shapes care-seeking behavior [2,7,16]. This supports the health capability model, where education and information expand a woman’s ability to navigate health systems and make informed delivery choices [7,16].

### Clinical and behavioral predictors in context

Other predictors align with established clinical understanding. ANC visits correlate strongly with CS likelihood, reflecting both improved clinical monitoring and the “gateway” effect of regular facility contact, which increases the probability of surgical referral [3,4,7]. Factors such as multiple births, terminated pregnancy history, and self-reported health status contributed moderately, consistent with their known association with elevated maternal and fetal risk [3,4].

Urban residence showed a moderate effect, which may be attenuated in some settings due to intra-urban inequities, fragmented referral pathways, and affordability barriers within urban health systems [1,16]. Conversely, the minimal influence of husband’s occupation and smoking status may reflect contextual specificity; spousal dynamics and tobacco use may be less salient determinants of CS in SSA or may be overshadowed by stronger structural factors such as wealth, education, and health system access [16].

### Methodological and policy implications

This study reinforces the value of machine learning in maternal health research, particularly for handling high-dimensional and imbalanced datasets such as the DHS. The application of SMOTE allowed for a more meaningful evaluation of model sensitivity to the minority CS outcome, moving beyond inflated accuracy metrics to better reflect discrimination performance [9,12]. The SHAP framework enhanced interpretability, transforming the model from a “black box” into an explanatory tool that reveals how specific features contribute to individual-level CS predictions [11,12].

However, the predictors identified are primarily sociobehavioral and economic proxies rather than direct clinical indicators. The absence of data on maternal body mass index, gestational age, intrapartum labor progression, fetal presentation, and obstetric complications limits the model’s clinical actionability. This highlights a fundamental limitation of DHS-based predictive modeling: while highly effective for identifying population-level inequities and structural drivers of service utilization, such surveys cannot substitute for facility-based clinical datasets in real-time risk stratification [20,26].

The findings primarily demonstrate methodological advantages of ML approaches rather than providing direct policy or clinical recommendations. The strong association between household wealth and CS utilization suggests. Given the nature of the dataset, these findings should not be interpreted as direct evidence for policy interventions but rather as insights into patterns captured in survey data. F [1,2,6]. The influence of education and media exposure highlights the role of targeted health communication strategies in promoting appropriate and informed use of CS [7,16]. Furthermore, ML-based tools such as the present framework could be integrated into antenatal screening systems to flag women at elevated risk, whether due to clinical proxies or socioeconomic vulnerability supporting earlier referral and delivery planning [10,26].

Model performance should be interpreted as pooled multicountry classification performance rather than evidence of uniform performance across individual SSA countries. Country-specific validation remains necessary.

## Strengths and limitations

This study utilized a large, nationally representative sample of 388,015 women from 10 Sub-Saharan African countries, enhancing generalizability. A range of modern machine learning algorithms was tested and compared, offering a robust evaluation of predictive performance.

SHAP values and feature importance techniques were used to ensure interpretability and transparency of model predictions. Statistical rigor was enhanced through repeated cross-validation, reporting of confidence intervals, and formal comparison against a logistic regression baseline. Balanced datasets using SMOTE enhanced the models’ sensitivity in detecting minority class events (i.e., CS deliveries). As a cross-sectional study, causal inferences cannot be drawn.

Potential recall bias may exist since the DHS data is self-reported and retrospective. Sensitivity analyses using alternative balancing strategies and country-stratified validation were not conducted; therefore, robustness across analytic assumptions and settings remains uncertain. Despite balancing techniques, the original class imbalance could influence model behavior.

Some potentially relevant clinical variables, such as maternal BMI, gestational age, and labor progress data, were not included, as they were unavailable in the DHS datasets. The absence of clinical variables such as BMI and gestational age may limit the model's ability to capture all medically relevant predictors, a challenge noted in similar ML-based obstetric studies [20].

Future research could explore the integration of LightGBM-based tools into antenatal care systems to provide real-time classification of utilization patterns, supporting clinicians in making timely, data-driven decisions [10].

## Conclusion

This study demonstrates that machine learning models, particularly LightGBM, can improve classification performance compared to logistic regression when applied to DHS data. Significant predictors included maternal age, child’s size at birth, ANC visits, maternal education, wealth index, and internet use. These insights can guide targeted interventions to ensure that CS procedures are used appropriately and equitably across populations. Integration of ML tools into maternal health classification of utilization patterns can enhance early risk identification and resource allocation, especially in low-resource settings. However, due to the cross-sectional design and absence of clinical predictors, the findings should not be interpreted as tools for clinical prediction or decision-making.

## Abbreviations

AdaBoost: Adaptive Boosting
ANC: Antenatal Care
ANN: Artificial Neural Network
AUC: Area Under the (ROC) Curve
BMI: Body Mass Index
CS: Cesarean Section
DHS: Demographic and Health Survey
DT: Decision Tree
EA: Enumeration Area
F1-score: Harmonic mean of Precision and Recall
IRB: Institutional Review Board
KNN: K-Nearest Neighbors
LightGBM: Light Gradient Boosting Machine
LMICs: Low and Middle-Income Countries
LR: Logistic Regression
ML: Machine Learning
MUAC: Mid-Upper Arm Circumference
PPS: Probability Proportional to Size
RF: Random Forest
ROC: Receiver Operating Characteristic
SD: Standard Deviation
SHAP: SHapley Additive exPlanations
SMOTE: Synthetic Minority Over-sampling Technique
SSA: Sub-Saharan Africa
WHO: World Health Organization
XGBoost: Extreme Gradient Boosting

## References

[1] Betran AP, Ye J, Moller A-B, Souza JP, Zhang J. Trends and projections of caesarean section rates: global and regional estimates. BMJ Glob Health. 2021;6(6):e005671. doi: 10.1136/bmjgh-2021-005671 34130991 PMC8208001

[2] Boerma T, Ronsmans C, Melesse DY, Barros AJD, Barros FC, Juan L, et al. Global epidemiology of use of and disparities in caesarean sections. Lancet. 2018;392(10155):1341–8. doi: 10.1016/S0140-6736(18)31928-7 30322584

[3] Sandall J, Tribe RM, Avery L, Mola G, Visser GH, Homer CS, et al. Short-term and long-term effects of caesarean section on the health of women and children. Lancet. 2018;392(10155):1349–57. doi: 10.1016/S0140-6736(18)31930-5 30322585

[4] Keag OE, Norman JE, Stock SJ. Long-term risks and benefits associated with cesarean delivery for mother, baby, and subsequent pregnancies: Systematic review and meta-analysis. PLoS Med. 2018;15(1):e1002494. doi: 10.1371/journal.pmed.1002494 29360829 PMC5779640

[5] Molina G, Weiser TG, Lipsitz SR, Esquivel MM, Uribe-Leitz T, Azad T, et al. Relationship Between Cesarean Delivery Rate and Maternal and Neonatal Mortality. JAMA. 2015;314(21):2263–70. doi: 10.1001/jama.2015.15553 26624825

[6] Vogel JP, Betrán AP, Vindevoghel N, Souza JP, Torloni MR, Zhang J, et al. Use of the Robson classification to assess caesarean section trends in 21 countries: a secondary analysis of two WHO multicountry surveys. Lancet Glob Health. 2015;3(5):e260-70. doi: 10.1016/S2214-109X(15)70094-X 25866355

[7] Fesseha N, Getachew A, Hiluf M, Gebrehiwot Y, Bailey P. A national review of cesarean delivery in Ethiopia. Int J Gynaecol Obstet. 2011;115(1):106–11. doi: 10.1016/j.ijgo.2011.07.011 21872239

[8] Cavallaro FL, Pembe AB, Campbell O, Hanson C, Tripathi V, Wong KL, et al. Caesarean section provision and readiness in Tanzania: analysis of cross-sectional surveys of women and health facilities over time. BMJ Open. 2018;8(9):e024216. doi: 10.1136/bmjopen-2018-024216 30287614 PMC6173245

[9] Rajkomar A, Dean J, Kohane I. Machine Learning in Medicine. N Engl J Med. 2019;380(14):1347–58. doi: 10.1056/NEJMra1814259 30943338

[10] Obermeyer Z, Emanuel EJ. Predicting the Future - Big Data, Machine Learning, and Clinical Medicine. N Engl J Med. 2016;375(13):1216–9. doi: 10.1056/NEJMp1606181 27682033 PMC5070532

[11] Islam MN, Mahmud T, Khan NI, Mustafina SN, Islam AKMN. Exploring Machine Learning Algorithms to Find the Best Features for Predicting Modes of Childbirth. IEEE Access. 2021;9:1680–92. doi: 10.1109/access.2020.3045469

[12] Sufriyana H, Husnayain A, Chen Y-L, Kuo C-Y, Singh O, Yeh T-Y, et al. Comparison of Multivariable Logistic Regression and Other Machine Learning Algorithms for Prognostic Prediction Studies in Pregnancy Care: Systematic Review and Meta-Analysis. JMIR Med Inform. 2020;8(11):e16503. doi: 10.2196/16503 33200995 PMC7708089

[13] Bari Antor M, Jamil AHMS, Mamtaz M, Monirujjaman Khan M, Aljahdali S, Kaur M, et al. A Comparative Analysis of Machine Learning Algorithms to Predict Alzheimer’s Disease. J Healthc Eng. 2021;2021:9917919. doi: 10.1155/2021/9917919 34336171 PMC8289609

[14] Talwar A, Lopez-Olivo MA, Huang Y, Ying L, Aparasu RR. Performance of advanced machine learning algorithms overlogistic regression in predicting hospital readmissions: A meta-analysis. Explor Res Clin Soc Pharm. 2023;11:100317. doi: 10.1016/j.rcsop.2023.100317 37662697 PMC10474076

[15] Ke G, Meng Q, Finley T, Wang T, Chen W, Ma W, et al. Lightgbm: A highly efficient gradient boosting decision tree. Advances in Neural Information Processing Systems. 2017;30.

[16] Anifowose O, Chummun B. A Panel Data Analysis of Determinants of Financial Inclusion in Sub-Saharan Africa (SSA) Countries from 1999 to 2024. JRFM. 2025;18(5):275. doi: 10.3390/jrfm18050275

[17] Manual DPSaHL. 2023. https://dhsprogram.com

[18] Program. DPTD. https://dhsprogram.com

[19] Fergus P, Hussain A, Al-Jumeily D, Huang D-S, Bouguila N. Classification of caesarean section and normal vaginal deliveries using foetal heart rate signals and advanced machine learning algorithms. Biomed Eng Online. 2017;16(1):89. doi: 10.1186/s12938-017-0378-z 28679415 PMC5498914

[20] Rahman S, Khan MdI, Satu MdS, Abedin MZ. Risk Prediction with Machine Learning in Cesarean Section: Optimizing Healthcare Operational Decisions. Intelligent Systems Reference Library. Springer International Publishing. 2020. 293–314. 10.1007/978-3-030-54932-9_13

[21] Chawla NV, Bowyer KW, Hall LO, Kegelmeyer WP. SMOTE: Synthetic Minority Over-sampling Technique. JAIR. 2002;16:321–57. doi: 10.1613/jair.953

[22] Learning Avitm. https://r2d3.us/visual-intro-to-machine-learning/

[23] S-l. Documentation. https://scikit-learn.org/stable/

[24] Lundberg SM, Lee SI. A unified approach to interpreting model predictions. Advances in Neural Information Processing Systems. 2017;30.

[25] Croft TN, Marshall AM, Allen CK, Arnold F, Assaf S, Balian S. Guide to DHS statistics. Rockville: ICF. 2018;645:292–303.

[26] Cavoretto PI, Candiani M, Farina A. Cesarean Delivery Uptake Trends Associated With Patient Features and Threshold for Labor Anomalies. JAMA Netw Open. 2023;6(3):e235436. doi: 10.1001/jamanetworkopen.2023.5436 36988960

